# A Neurophysiological Study of Musical Pitch Identification in Mandarin-Speaking Cochlear Implant Users

**DOI:** 10.1155/2020/4576729

**Published:** 2020-07-22

**Authors:** Jieqing Cai, Yimeng Liu, Minyun Yao, Muqing Xu, Hongzheng Zhang

**Affiliations:** Department of Otolaryngology Head & Neck Surgery, Zhujiang Hospital, Southern Medical University, Guangzhou, China 510282

## Abstract

Music perception in cochlear implant (CI) users is far from satisfactory, not only because of the technological limitations of current CI devices but also due to the neurophysiological alterations that generally accompany deafness. Early behavioral studies revealed that similar mechanisms underlie musical and lexical pitch perception in CI-based electric hearing. Although neurophysiological studies of the musical pitch perception of English-speaking CI users are actively ongoing, little such research has been conducted with Mandarin-speaking CI users; as Mandarin is a tonal language, these individuals require pitch information to understand speech. The aim of this work was to study the neurophysiological mechanisms accounting for the musical pitch identification abilities of Mandarin-speaking CI users and normal-hearing (NH) listeners. Behavioral and mismatch negativity (MMN) data were analyzed to examine musical pitch processing performance. Moreover, neurophysiological results from CI users with good and bad pitch discrimination performance (according to the just-noticeable differences (JND) and pitch-direction discrimination (PDD) tasks) were compared to identify cortical responses associated with musical pitch perception differences. The MMN experiment was conducted using a passive oddball paradigm, with musical tone C4 (262 Hz) presented as the standard and tones D4 (294 Hz), E4 (330 Hz), G#4 (415 Hz), and C5 (523 Hz) presented as deviants. CI users demonstrated worse musical pitch discrimination ability than did NH listeners, as reflected by larger JND and PDD thresholds for pitch identification, and significantly increased latencies and reduced amplitudes in MMN responses. Good CI performers had better MMN results than did bad performers. Consistent with findings for English-speaking CI users, the results of this work suggest that MMN is a viable marker of cortical pitch perception in Mandarin-speaking CI users.

## 1. Introduction

Hair cells (HCs) in the cochlea play a critical role in converting mechanical sound waves into electric signals for hearing [1]. HCs are vulnerable for multiple damages, including noise, different ototoxic drugs, inflammation, and aging [2–8]. In mammals, damaged HCs cannot be spontaneously regenerated [9–13]; thus, sensorineural deafness is permanent once HCs are damaged. Cochlear implants (CIs) can partially replace the function of HCs and are the primary clinical therapeutic devices for patients with severe and profound sensorineural deafness by far. According to incomplete statistics, more than 500,000 deaf patients worldwide have recovered hearing through cochlear implantation [14]. The basic working principle of a CI is as follows: the microphone picks up sound signals and converts them to digital sound waves, then transmits the electrical signals to the speech processor, which encodes signals and generates electrical pulses corresponding to different electrodes to directly stimulate the auditory nerve fibers in different regions of the cochlea [15]. Although electrical hearing is extremely degraded and unnatural, most CI users can achieve good speech recognition in quiet conditions and meet the fundamental requirements of everyday verbal communication [16]. Nevertheless, many CI users show far from satisfactory performance in challenging listening tasks, such as tone recognition in Mandarin Chinese, speech recognition in noisy conditions or with competing sound sources, and music perception [17–19]. Music has more complex, abstract, and varied acoustic characteristics than does speech; its four primary elements are rhythm, pitch, volume, and timbre. The musical perception ability of CI users has been assessed according to rhythm, pitch, melody, and timbre using various test platforms (e.g., the clinical assessment of music perception test (CAMP) and the musical sounds in cochlear implants perception test (MuSIC)) [20–22]. Many early behavioral studies revealed equivalent musical rhythm recognition in CI users and normal-hearing (NH) listeners, but poorer pitch, melody, and timbre recognition in CI users [20, 22–27]. Behavioral data obtained with the just-noticeable differences (JND) and pitch-direction discrimination (PDD) tasks are regarded as suitable for the measurement of pitch discrimination (i.e., definition of pitch perception thresholds) during peripheral auditory processing [18, 22, 28, 29].

Behavioral tests were used to evaluate the music perception of CI users in almost all early studies. However, with the development of electrophysiological technology, cortical auditory processing in music discrimination can be further understood by mismatch negativity (MMN) assessment. MMN is a component of the endogenous event-related potential, an electroencephalographic (EEG) response evoked by the insertion of any discernible deviation in a series of standard stimuli [30]. MMN is an electrophysiological index of the brain's automatic processing of sensory information, independent of listening tasks and selective attention. It can be elicited by a stimulus with deviation of any distinguishable acoustic characteristic, such as frequency, intensity, duration, or timbre, in pure tones, speech, and music [31–33]. Previous studies revealed that MMN is a viable, objective, and noninvasive measure of auditory discrimination [34].

Recently, MMN has also been used to evaluate speech recognition and rehabilitation in CI users. Turgeon et al. [35] measured MMN using a two-deviant oddball paradigm based on speech syllables (/da/, /ba/, and /ga/), revealing a significant positive correlation between the amplitude of MMN and the speech recognition score. These findings suggest that MMN is an objective measure of speech recognition ability in CI users. Another study showed that the MMN amplitude in a vowel-duration identification task was similar in children who had worn CIs for 4 months and in their NH counterparts, suggesting the existence of auditory cortex plasticity [36]. In addition to its extensive application in speech recognition studies, researchers have extended MMN to the measurement of music (e.g., rhythm, pitch, timbre, melody, and chord) perception [33]. The relationship between preattention and rhythm processing was studied by changing the rhythm structure [37]. In early studies, multifeature paradigms were used with MMN to examine CI users' perception of music timbre, intensity, and rhythm. The results showed that the latency of MMN was prolonged and its amplitude was reduced, reflecting impaired music perception ability, in CI users compared with NH listeners [38–41].

Several studies have used MMN to explore neurophysiological responses related to musical pitch discrimination in CI users. [42] used a multifeature MMN paradigm with deviant stimuli in different acoustic dimensions (i.e., frequency, intensity, and duration) to assess music perception in CI users. They found that the latency of MMN decreased and its amplitude increased with the increased frequency of deviant stimuli for NH subjects, but those changes were irregular, reflecting impaired pitch perception, for CI users. Another study showed that MMN could be evoked by as few as two and four semitones of pitch deviation, with significantly prolonged MMN latency and reduced amplitude in CI users compared with NH subjects [39]. In previous research, behavioral tests have been used to obtain a perceptual threshold for timbre, with stimuli for MMN recording (including suprathreshold and subthreshold stimuli) set according to individual behavioral thresholds [43]. However, few studies have investigated MMN responses and their correlations with musical pitch discrimination thresholds using behavioral tests with Mandarin-speaking CI users and NH listeners.

Most active, ongoing neurophysiological studies of music perception do not involve CI users who speak tonal languages. Mandarin is the most widely spoken tonal language; lexical meanings are conveyed through four tone patterns with different pitch-change contours. The tones of Mandarin are classified according to the pattern of fundamental frequency (F0) variation and the absolute frequency of pitch and are distinguished predominantly by changes in the F0 contour and duration [44]. Musical pitch, a perceptual sound property, depends mainly on F0 and harmonic components. Several behavioral studies have demonstrated that pitch and lexical tone perception are correlated and have similar underlying mechanisms in CI-based electric hearing [19, 45, 46]. Therefore, accurate pitch discrimination is crucial for the understanding of tonal languages, such as Mandarin Chinese.

Mandarin-speaking CI users must process tonal information in everyday communication, but whether this processing has any impact on the auditory processing of musical pitch is unclear. In addition, clinical data regarding the characteristics of MMN in pitch perception in Mandarin-speaking CI users are insufficient. Therefore, the present study is aimed at providing evidence for the neurophysiological mechanisms underlying musical pitch discrimination in Mandarin-speaking CI users. Specifically, MMN characteristics (e.g., amplitudes and latencies) and their relationships to the minimal identification thresholds of pitch differences in JND and PDD tasks were examined. We hypothesized that automatic cortical processing related to musical pitch perception would be reflected in the MMN responses of CI users. Compared with NH listeners, CI users with higher behavioral thresholds had worse elicited performance according to EEG measures. In addition, MMN responses marked differences in cortical responses between CI users with good and poor musical pitch identification performance.

## 2. Methods

### 2.1. Subjects

Eleven CI users (seven females and four males) aged 10–40 years participated in this study. These subjects were recruited from Zhujiang Hospital, Southern Medical University. One participant had bilateral CIs (only one side was used during the test), and the others had unilateral CIs. The CI users' demographic information is shown in Table 1. All CI-using participants were right-handed, with normal verbal communication ability, normal mental and intellectual development, and no formal musical training; patients with auditory neuropathy and neurological diseases were excluded. All subjects were required to complete behavioral and EEG testing. The CI users were divided into good and poor performance groups according to musical tone discrimination test results. The control group consisted of 12 NH subjects (6 females and 6 males) aged 19–25 years with pure tone audiometry thresholds < 25 dB HL at octave frequencies of 0.25–8 kHz. NH subjects had no history of otitis media or psychiatric or neurological disease. The Ethics Committee of Zhujiang Hospital, Southern Medical University, approved this study; the ethical approval number was 2017-EBYHZX-001. All subjects participated in the study voluntarily. Each participant provided written informed consent, and participants under the age of 16 gave written informed consent from their parents.

### 2.2. Stimuli

The test stimuli were synthetic complex tones. Each tone consisted of F0 and two harmonics with amplitudes attenuated by 20% per octave (first harmonic, 80% amplitude; second harmonic, 60% amplitude). The duration of each stimulus was 500 ms, including 25 ms each for onset and offset ramping to reduce sudden spectral shift. The intensity of the sound stimuli was normalized using the root-mean-square method. For the MMN test, the stimuli were the musical tones C4 (262 Hz), D4 (294 Hz, 2-semitone pitch interval from C4), E4 (330 Hz, 4-semitone interval), G#4 (415 Hz, 8-semitone interval), and C5 (523 Hz, 12-semitone interval).

The MMN experiment was conducted using a passive oddball paradigm, with tone C4 presented as the standard (~87% probability of occurrence) and tones D4, E4, G#4, and C5 presented as deviants (~13% probability of occurrence). The interstimulus interval (ISI) was 600 ms, and the stimuli were played in a pseudorandom sequence; at least three standard stimuli were presented between two deviant stimuli. The test comprised four blocks of stimuli (total, 2848 standard stimuli (4 × 712) and 420 deviant stimuli (105 each of D4, E4, G#4, and C5)).

The tests were carried out in a sound-insulated, electrically shielded room, with <30 dBA background noise. Experimental auditory stimuli were presented through a loudspeaker (model S1000MA; Edifier) placed 1.2 m in front of the subjects at a seated ear level. The sound intensity was approximately 65 dBA. During the MMN experiment, the subjects were instructed to sit comfortably and to optionally watch the silent films presented, to pay no attention to the stimuli, to keep quiet and awake, and to reduce limb movements and blinking. The four test blocks were delivered to each subject in a counterbalanced sequence, with approximately 3 min rest between blocks.

### 2.3. Psychoacoustic Testing

Before testing, all participants filled out a questionnaire on their music experience, which was designed for this study. We selected three questionnaire items (on the frequencies with which respondents listened to music and sang and on their degree of enjoyment of music; see the appendix for details) for the evaluation of music experience. Scores ranged from 1 (“not at all”) to 10 (“very often”). CI users responded according to their postimplantation situations, and NH listeners responded according to their usual situations.

All participants performed the JND and PDD tasks using music perception evaluation software [47]. Before testing, they conducted preliminary runs to become familiarized with the test materials and procedures. Three alternative-forced choices with a three-down, one-up adaptive tracking procedure were used for the JND task, with an initial pitch interval of 12 semitones. The base frequency of the reference tone was C4. Three tones (one target and two references) were played randomly in each trial, with an ISI of 1 s. Participants were asked to identify the pitch that sounded “different.” The test was ended when the participant attained 12 reversals or 3 consecutive correct discriminations at a 1-semitone pitch interval. The mean of the last six reversals was calculated as the final threshold of pitch difference discrimination. The PDD task was implemented using a two-alternative-forced choice approach, a target tone and a reference tone. Subjects were asked to choose the tone with the higher pitch. The PDD test procedure and threshold calculation were the same as for the JND test.

### 2.4. EEG Recording

EEG data were obtained with a SynAmps amplifier (NeuroScan, Charlotte, NC, USA) using a 64-electrode cap placed according to the international 10-20 system. The reference electrodes were placed on the contralateral mastoids (M1, M2) and the nasal tip of each subject. The vertical electrooculogram was monitored by an external electrode placed below the left eye. The EEG data were recorded with a band-pass filter setting of 0.1–100 Hz and a sampling rate of 500 Hz. Impedance in each electrode was kept below 5 k*Ω* before data acquisition. The electrodes located near the CI transmission coil were not used for CI-using participants.

### 2.5. EEG Data Analysis

EEG data were analyzed with EEGLAB 14.1.1 [48] in Matlab 2015b (MathWorks, Natick, MA, USA). First, the data were rereferenced using the contralateral mastoid signals. Then, continuous EEG data were filtered using a 50 Hz notch and a band-pass filter (1–30 Hz). EEG signals exceeding ±50 *μ*V and with nonstereotyped artifacts (<10% of individual subjects' datasets) were regarded as bad blocks and were removed before further analysis. Data for electrodes located near CI transmission coils were interpolated using data from four adjacent electrodes. Next, infomax independent component analysis was performed for artifact (e.g., eye blinks, horizontal eye movement, electrocardiographic activity, and CI electrical stimulation) correction. After artifact removal, each epoch was selected between 100 ms prestimulus and 500 ms poststimulus and corrected with the baseline of the prestimulus time window, and event-related potentials were calculated by temporal averaging of epochs with the same type of stimulus.

MMN waveforms were obtained by subtracting the response to the standard (C4) from the response to each of the four deviants (E4, G#4, C5, and D4). Grand-average difference waveforms were, respectively, computed for the four deviants in the CI and NH groups. The Fz electrode in the frontocentral region was used for MMN analysis, the largest negative MMN peak is typically obtained at Fz [31, 49], and the neural response was consistent in different subjects. The peak amplitude and latency of MMN responses were calculated within the 156–236 ms window for the CI group and the 130–210 ms window for the NH group. Window selection was based on previous MMN studies [49, 50] and average MMN results for deviants in this experiment.

### 2.6. Statistical Analysis

Statistical analyses were implemented with SPSS 20.0. (PSS Inc., Chicago, IL, USA). Two independent-sample nonparametric tests and analysis of variance (ANOVA) were used to examine differences in JND and PDD task performance, age, and music experience between CI users and NH listeners. Repeated-measures ANOVA was conducted with the grand-average MMN amplitudes and latencies, with two main factors: listening status (CI and NH) and deviant type (E4, G#4, C5, and D4).

## 3. Results

### 3.1. Musical Pitch Recognition

The two groups were matched in terms of age and music experience (*p* > 0.05; Table 2). The two groups of CI users with good and poor performance were also matched in terms of music experience (*p* > 0.05). The mean thresholds for CI users in the JND and PDD tasks were 3.1 ± 1.4 (range, 1.0–5.0) semitones and 4.2 ± 4.2 (range, 1.0–15.8) semitones, respectively. For NH listeners, these thresholds were 1.2 ± 0.8 (range, 1.0–3.8) and 1.9 ± 1.4 (range, 1.0–6.8) semitones, respectively. The thresholds were significantly higher for CI users than NH controls (JND task: *Z* = –3.698, *p* < 0.001; PDD task: *Z* = –2.167, *p* = 0.030; Table 2). According to the JND and PDD task results, CI users with minimum pitch discrimination ability ≤ 4 semitones were allocated to the good performance group (*n* = 5), and those with >4-semitone discrimination ability were allocated to the poor performance group (*n* = 6).

### 3.2. MMN

#### 3.2.1. NH Listeners vs. CI Users

Two-way repeated-measures ANOVA was conducted with two main factors: (1) pitch differences between the standard and deviants (12, 8, 4, and 2 semitones) and (2) listener group (NH listeners and CI users). The two-way repeated-measures ANOVA was conducted with data from seven CI users and seven NH listeners, as only seven CI users finished the experiments under four conditions with distinct pitch changes. The main effect of pitch difference on the MMN amplitude was observed in NH listeners and CI users (*F* (3, 7) = 7.055, *p* < 0.05), but no main effect was found for the listener group or interaction between these two factors. One-way ANOVA was performed to assess the effects of pitch difference in NH listeners and CI users. The MMN amplitude increased significantly with the pitch difference in NH listeners (*F* (3, 12) = 6.978, *p* < 0.01). Such increases were observed for large pitch changes (12, 8, and 4 semitones) in the 11 CI users (*F* (2, 11) = 5.854, *p* < 0.05).

**Figure 1:**
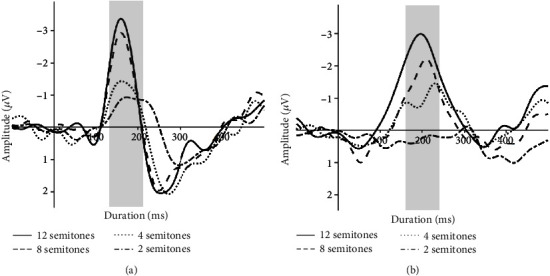
Grand-average MMN waveforms for distinct pitch differences for NH listeners (a) and CI users (b) at electrode Fz. Gray shading indicates time windows used to calculate amplitudes and latencies ((a) 130–210 ms; (b) 156–236 ms).

**Figure 2:**
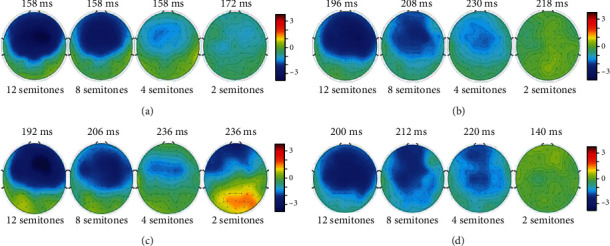
Contour maps of MMN amplitude for grand-average differences in waveforms for NH listeners (a) and CI users (b) and CI users with good (c) and poor (d) performance. Topological distributions are displayed at latencies with peak MMN amplitudes in each plane.

Two (NH listeners and CI users) by four (pitch differences) repeated-measures ANOVA was used to further assess the effect of pitch difference on MMN latency. The analysis revealed an interaction between the subject group and pitch differences (*F* (3, 7) = 7.542, *p* < 0.05). The two-way interaction was characterized by significant differences between NH listeners and CI users in MMN latencies (*F* (1, 7) = 7.945, *p* < 0.05), which were shorter for a given pitch difference among NH listeners than among CI users.  Figure 1 shows the MMN responses to 12-, 8-, 4-, and 2-semitone pitch differences in NH listeners and CI users. The peak amplitudes and latencies of MMN waveforms under different conditions are summarized in Table 3.  Figure 2 shows the topological distribution of latencies at peak amplitudes in NH listeners and CI users.

#### 3.2.2. CI Users with Good vs. Poor Performance

**Figure 3:**
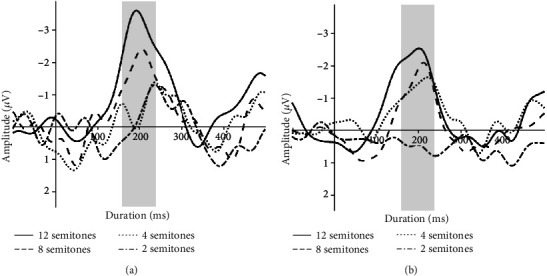
Grand-average MMN waveforms for distinct pitch differences for CI users with good (a) and poor (b) performance at electrode Fz. Gray shading indicates the time window used to calculate amplitudes and latencies (156–236 ms).

Figure 3 illustrates MMN waveforms according to pitch changes in CI users with good and poor performance, and Table 4 provides detailed MMN amplitude and latency results. Separate plots for CI users with good and poor performance are presented in Figures 2(c) and 2(d). Two-way ANOVA was conducted to assess whether MMN waveforms elicited by pitch differences were affected by CI users' pitch discrimination performance. The statistical analysis excluded data from 2-semitone pitch changes because only two CI users with poor performance participated in the test under this condition. Pitch changes had a main effect on the MMN amplitude in CI users with good and poor performance (*F* (3, 5) = 10.904, *p* < 0.05); the amplitude increased significantly with the difference between standard and deviant tones. Pitch changes had no significant effect on MMN latency in the two CI groups.

### 3.3. Correlations in CI Users

**Figure 4:**
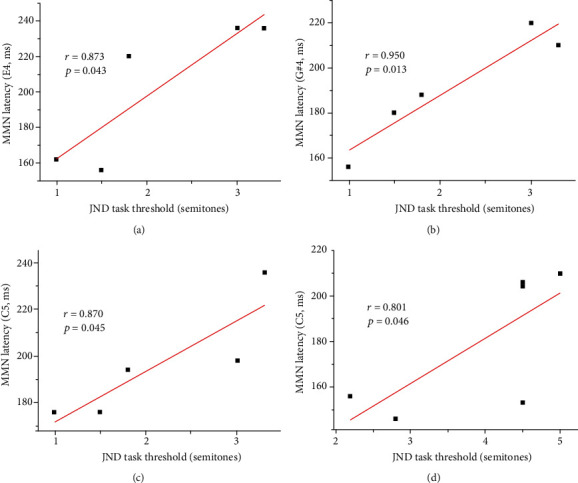
Correlations between JND task thresholds and MMN latency by pitch deviation in CI users with good (a–c) and poor (d) performance.

Bivariate correlation analysis revealed positive correlations between the JND task threshold in well-performing CI users and MMN latency for E4 (*r* = 0.873, *p* = 0.043), G#4 (*r* = 0.950, *p* = 0.013), and C5 (*r* = 0.870, *p* = 0.045), but no correlation with MMN amplitude. For poorly performing CI users, the JND task threshold was correlated positively with the MMN latency for C5 (*r* = 0.801, *p* = 0.046). No correlation was observed between MMN latency or amplitude and the PDD task threshold, music experience, duration of deafness, CI experience, or age of cochlear implantation (Figure 4, Table 5).

## 4. Discussion and Conclusions

HCs in the inner ear cochlea play an important role for hearing [1, 51]. In mammal's inner ear cochlea, HCs are sensitive for multiple stresses and easy to be damaged. Thus, most of the sensorineural deafness induced by gene mutation, noise, different ototoxic drugs, inflammation, or aging are caused by the HC loss [52–57]. However, the mammals only have very limited HC regeneration ability; most of the damaged HCs cannot be spontaneously regenerated, which make the HC loss and hearing loss to be irreversible [58–62]. CIs are the most efficient clinical therapeutic devices for sensorineural deafness patients, and recent studies have shown that application of CI-based electric acoustic stimulation together with multiple biomaterials also can promote the differentiation of neural stem cell [63–66] and promote maturation of spiral ganglion neuron [67–70]. However, the neurophysiological study of musical pitch identification in CI users is still lacking in the hearing research field. The aim of the present study was to investigate neurophysiological responses relevant to musical pitch discrimination in CI users and NH listeners using an oddball paradigm with four deviant stimuli. Relationships between MMN response features and behavioral results in CI individuals with distinct musical pitch discrimination ability were also investigated.

CI users generally had difficulty discriminating musical pitch changes compared with NH controls. The neurophysiological data also demonstrated that CI users had more difficulty with preattentive discrimination of musical pitch than did NH listeners, reflected in significantly prolonged MMN latencies. Researchers have suggested that poorer musical pitch perception in CI users compared with NH listeners is due to the lack of adequate temporal and spectral cues transmitted by the CI device [46, 71]. The limited number of channels and crude spectral-temporal cues lead to poor spectral resolution, rendering the accurate comprehension of musical tones difficult [27]. In addition, the frequency information carried by the electrodes likely does not match the actual frequency produced in the cochlea, which degrades pitch perception ability [72]. Moreover, the neurophysiological alterations and crossmodal plasticity of the auditory center that generally accompany long-term deafness may interfere with auditory processing [73]. As in early studies [31], MMN responses were evoked mainly in the frontal area in this study. The topological distribution of MMN responses implies weaker preattentive auditory perception in CI users compared with NH listeners.

Early studies showed that CI users' pitch thresholds ranged from 1 to 24 semitones [24, 28]; we obtained similar behavioral results, with a range of 1.0–15.8 semitones and a high degree of variability among individuals. To better evaluate CI users' performance, we divided into good and poor performance groups according to JND and PDD task results. MMN responses have been proven to be objective predictors of musical pitch perception ability, with amplitudes and latencies sensitive to differences between deviant and standard stimuli [34]. Thus, we used MMN responses to compare preattentive cortical activation between well-performing and poorly performing CI users. The marginally significant increase in MMN amplitude for good performers relative to poor performers reflects consistency between the auditory cortical responses and behavioral results. In addition, well-performing, but not poorly performing, CI users showed MMN responses to 2-semitone pitch differences, reflecting the human brain's auditory plasticity after cochlear implantation and hearing rehabilitation. The behavioral results for all CI users did not reflect such sensitivity to 2-semitone differences; they reflect integrated auditory perception with peripheral and central stages, whereas MMN responses reflect only automatic preattentive pitch discrimination ability at the central level [74]. The MMN responses to 2-semitone pitch differences also support MMN as an effective cortical response predictor in the development of auditory training strategies and parameter settings for CI devices [75, 76].

This study revealed a positive correlation between the latency of MMN waveforms in CI users and the JND, but not PPD, task threshold. Some previous studies also demonstrated positive correlations between MMN responses and speech recognition scores [35, 39]. These results show that MMN responses are better for the identification of pitch difference discrimination ability in CI users. The lack of correlation with PDD results may arise from differences in behavioral test characteristics. The JND task requires subjects to detect differences in musical pitch, whereas the PDD task requires them to distinguish the highest of two pitches and to identify the contour of pitch changes in a successive pitch sequence. MMN responses are elicited when subjects preattentively detect differences between standard and deviant stimuli, which does not involve complex pitch recognition or advanced brain function. These properties may explain the correlation of these responses only with JND task performance. To further explore correlations between cortical responses and more complex cognitive behavioral results (i.e., of the PDD task), we will use an active experimental paradigm to examine EEG components in late latencies (e.g., P300 and N400) [77] in future studies.

Importantly, this work examined the musical pitch discrimination abilities of native Mandarin speakers using both behavioral and neurophysiological tests. Mandarin-speaking CI users may have advantages in pitch information identification due to their long-term exposure to the tonal language environment. Early studies supported the similarity of the perceptual mechanism underlying the perception of Mandarin tones and musical pitches with electric stimulation [19, 78]. Consistent with previous findings [39, 42, 79, 80], we found that these CI users were able to distinguish musical pitches under a preattentive auditory condition. Furthermore, even slight (e.g., 2-semitone) pitch differences evoked MMN responses in Mandarin-speaking CI users with good behavioral performance. These findings suggest that the abilities to identify musical pitches and Mandarin tones are correlated. Better ability to discriminate Mandarin tones appears to facilitate the identification of musical pitch differences and vice versa.

In conclusion, this study evaluated the music pitch discrimination performance of Mandarin-speaking CI users and NH listeners using behavioral and MMN measures. MMN response latency was correlated strongly with the JND task pitch discrimination threshold in CI users. The CI users with good JND task performance had enhanced MMN amplitudes and shorter latencies compared with CI users with poor JND task performance. Consistent with findings from studies of English-speaking CI users, the findings from this work support the feasibility of MMN use for the evaluation of musical pitch identification performance and its potential to aid outcome evaluation following cochlear implantation and hearing rehabilitation among CI users.

## Figures and Tables

**Table 1:** Clinical information of CI users. LVAS: large vestibular aqueduct syndrome.

Subject	Age	Deafness duration (years)	CI experience (years)	Etiology	Implant ear	Type of CI	Classification of deafness
CI01	19	16	1	LVAS	Left	Nucleus RE24	Postlingual
CI02	11	2	8.6	Congenital	Right	Nucleus RE24	Prelingual
CI03	14	10	0.3	LVAS	Left	AB HiRes 90K	Postlingual
CI04	14	12	0.3	LVAS	Right	Nucleus CI512	Postlingual
CI05	21	5	16	Congenital	Right	Nucleus R24	Prelingual
CI06	20	3	17	Congenital	Right	Nucleus R24	Prelingual
CI07	24	8	16	Congenital	Left	Nucleus RE24	Prelingual
CI08	40	20	Right: 11; left: 1	Progressive	Both	Nucleus R24	Postlingual
CI09	10	3	7	Congenital	Right	Nucleus RE24	Prelingual
CI10	25	7	5.8	Unknown	Right	Nucleus RE24	Postlingual
CI11	23	2	1.5	Unknown	Right	Nucleus RE24	Postlingual

**Table 2:** Statistical results for age, music experience, and pitch discrimination scores for the two study groups.

Group	JND (semitone) (SD)	PDD (semitone) (SD)	Age (year) (SD)	Music experience (SD)
CI users	3.1 (1.4)	4.2 (4.2)	20.1 (8.4)	6.73 (2.33)
NH controls	1.2 (0.8)	1.9 (1.4)	21.3 (1.7)	7.54 (1.71)
*Z*/*F*	-3.698	-2.167	0.223	0.965
*p*	<0.001	0.030	0.646	0.337

JND: just-noticeable difference task; PDD: pitch-direction discrimination task; SD: standard deviation.

**Table 3:** Statistical results of the MMN amplitude and latency (average ± standard deviant) for 12-semitone, 8-semitone, 4-semitone, and 2-semitone pitch changes in NH listeners and CI users.

	Pitch changes	Amplitude (*μ*V)	Latency (ms)
Normal hearing	12 semitones	−3.77 ± 0.55	153.83 ± 4.52
	8 semitones	−3.11 ± 0.41	159.67 ± 3.19
	4 semitones	−2.14 ± 0.41	165.50 ± 6.34
	2 semitones	−1.51 ± 0.24	184.67 ± 5.75
Cochlear implants	12 semitones	−3.83 ± 0.54	187.09 ± 7.92
	8 semitones	−2.78 ± 0.54	197.27 ± 8.74
	4 semitones	−2.42 ± 0.34	204.18 ± 10.04
	2 semitones	−0.69 ± 0.43	178.00 ± 10.34

**Table 4:** MMN amplitude and latency (mean ± standard deviation) according to pitch change in CI users with good and poor performance.

	Pitch changes	Amplitude (*μ*V)	Latency (ms)
Cochlear implants (good performers)	12 semitones	−4.23 ± 0.94	196.00 ± 9.81
	8 semitones	−2.95 ± 0.82	190.80 ± 10.11
	4 semitones	−2.30 ± 0.06	202.00 ± 15.94
	2 semitones	−1.74 ± 0.87	209.00 ± 16.26
Cochlear implants (poor performers)	12 semitones	−3.50 ± 0.56	179.67 ± 11.13
	8 semitones	−2.64 ± 0.71	202.67 ± 13.23
	4 semitones	−2.51 ± 0.63	206.00 ± 12.68
	2 semitones	−0.27 ± 0.35	165.60 ± 7.72

**Table 5:** Correlations between pitch discrimination thresholds and MMN amplitude and latency by pitch change in CI users with good and poor performance. Significant correlations (*p* < 0.05) are presented in bold.

MMN		Pitch changes	JND		PDD	
			*r*	*p*	*r*	*p*
Cochlear implants (good performers)	Amplitude (*μ*V)	12 semitones	-0.132	0.832	0.541	0.347
		8 semitones	-0.151	0.808	0.351	0.562
		4 semitones	-0.806	0.100	-0.100	0.873
	Latency (ms)	12 semitones	0.870	**0.045**	0.153	0.806
		8 semitones	0.950	**0.013**	-0.202	0.774
		4 semitones	0.873	**0.043**	-0.443	0.455
Cochlear implants (bad performers)	Amplitude (*μ*V)	12 semitones	0.577	0.231	-0.386	0.450
		8 semitones	0.213	0.686	0.159	0.764
		4 semitones	0.152	0.774	0.005	0.992
	Latency (ms)	12 semitones	0.801	**0.046**	-0.631	0.179
		8 semitones	0.277	0.595	-0.556	0.242
		4 semitones	0.462	0.356	-0.679	0.138

## Data Availability

The data used to support the findings of this study are available from the corresponding author upon request.
